# Transcriptome Profiling Analysis Reveals Changes in the Antioxidant Defense System, Morphology, and Gene Expression in the Gills of *Macrobrachium nipponense* Caused by Alkalinity Exposure

**DOI:** 10.3390/ijms26094321

**Published:** 2025-05-01

**Authors:** Shubo Jin, Yuefan Zhang, Hongtuo Fu, Wenyi Zhang, Hui Qiao, Yiwei Xiong, Sufei Jiang

**Affiliations:** 1 Key Laboratory of Freshwater Fisheries and Germplasm Resources Utilization, Ministry of Agriculture and Rural Affairs, Freshwater Fisheries Research Center, Chinese Academy of Fishery Sciences, Wuxi 214081, Chinazhangwy@ffrc.cn (W.Z.);; 2 Key Laboratory of Mariculture & Stock Enhancement in North China’s Sea, Ministry of Agriculture and Rural Affairs, Dalian Ocean University, Dalian 116023, China

**Keywords:** *Macrobrachium nipponense*, alkaline exposure, gill, immune response, energy metabolism

## Abstract

The median lethal concentration value of alkalinity tolerance for *Macrobrachium nipponense* over 96 h is only 14.42 mmol/L with a safety value of 4.71 mmol/L, which is insufficient to perform the aquaculture program in a water environment with high alkalinity. Thus, the present study aims to explore the effects of alkalinity exposure on the gills of *M. nipponense* through identifying the changes in antioxidant enzymes, morphology, and gene expressions after 1 day, 4 days, and 7 days of exposure under an alkalinity of 10 mmol/L. The activities of MDA, GSH-PX, CAT, T-AOC, and Ca^2+^Mg^2+^-ATPase are significantly stimulated by 62.6%, 6.57%, 32.1%, 33.3%, and 14.9%, compared to those from Day 0 (control group), indicating that these antioxidant enzymes play essential roles in the protection of prawns from the damage of reactive oxygen species caused by alkalinity exposure. In addition, alkalinity exposure results in an increase in the hemolymph vessels, affecting the normal respiratory function of the gills. Transcriptome profiling analysis reveals that short-term alkali exposure (4 days) does not result in significant changes in gene expression in the present study. Furthermore, metabolic pathways, biosynthesis of amino acids, amino sugar and nucleotide sugar metabolism, lysosomes, glycolysis/gluconeogenesis, and phagosomes represent the main enriched metabolic pathways of differentially expressed genes (DEGs) between Day 4 and Day 7. Biosynthesis of amino acids, lysosomes, and phagosomes are immune-related metabolic pathways, while amino sugar and nucleotide sugar metabolism and glycolysis/gluconeogenesis are energy metabolism-related metabolic pathways, indicating that the processes of immune response and energy metabolism play essential roles in the response to alkalinity exposure in *M. nipponense*. Thus, the DEGs from these metabolic pathways are considered as candidate genes involved in the regulation of alkaline acclimation in *M. nipponense*. The present study provides valuable evidence for analysis of the adaptive mechanism when exposed to alkalinity, contributing to the survival rate and aquaculture of this species under water environments with high alkalinity.

## 1. Introduction

Saline–alkaline water resources are widely distributed in inland areas, representing a global water resource with a low yield of both aquatic animals and crops. The saline–alkaline water resources in China have reached 9.91 × 10^7^ hectares, ranking third in the world and accounting for 10% of the world’s total saline–alkaline water resources. The main saline–alkaline water resources in China are widely distributed in the northeastern, northern, and southwestern parts of China, and coastal areas. Compared to those of seawater, saline–alkaline water has the characteristics of a high pH, high carbonate alkalinity, high water mineralization, diverse hydrochemical types, complex ion composition, lack of constant ion ratio, and poor water quality buffering capacity, meaning it is not suitable for use as drinking water for humans and animals or as irrigation water for agriculture [1,2]. Economically aquatic animals with a weak alkalinity tolerance cannot normally produce and reproduce in such water resources, leading to low aquaculture yields in such areas. Although China has abundant saline–alkaline water resources, the utilization rate of such water resources is less than 2%, and they have not been effectively utilized [3,4].

Alkalinity tolerance varies greatly between different aquatic animals. Many aquatic animals have been identified their alkalinity tolerance. For example, the median lethal concentration (LC_50_) values for 12 h, 24 h, 48 h, 72 h, and 96 h in *Tribolodon brandti Dybowski* were 98.79 mmol/L, 89.31 mmol/L, 79.34 mmol/L, 78.45 mmol/L, and 68.44 mmol/L, respectively, with a safety value of 18.79 mmol/L [5]. The LC_50_ values of *Misgurnus mohoity*, *Paramisgurnus dabryanus*, *Triplophysa dalaica*, and *Colossoma brachypomum* for 96 h were 88.83 mmol/L, 155.2 mmol/L, and 120.0 mmol/L, with safety values of 18.77 mmol/L, 23.66 mmol/L, and 36.30 mmol/L, respectively [6]. The LC_50_ values in *Colossoma brach ypomum* were 83.25 mmol/L for 24 h, 56.99 mmol/L for 48 h, and 45.70 mmol/L for 96 h [7]. Previous publications have also identified alkalinity tolerance in many crustaceans. The LC_50_ values in *Eriocheir sinensis* were 110.43 mmol/L for 24 h, 90.50 mmol/L for 48 h, 75.78 mmol/L for 72 h, and 69.74 mmol/L for 96 h with a safety value of 18.25 mmol/L [8]. The LC_50_ values in juvenile *M. rosenbergii* for 24 h, 48 h, 72 h, and 96 h were 51.02 mmol/L, 37.07 mmol/L, 27.19 mmol/L, and 21.54 mmol/L, respectively [9]. The LC_50_ values in *Litopenaeus vannamei* for 24 h, 48 h, and 96 h were 12.92 mmol/L, 11.16 mmol/L, and 10.67 mmol/L with a safe alkali concentration of 2.50 mmol/L, under pH values of 8.03–8.37 [10]. Fish species generally showed stronger alkaline resistance than that of crustaceans.

*Macrorachium nipponense* is an economically important freshwater prawn species in many Asian countries, including China, Japan, Korea, Vietnam, and Myanmar, which has gained significant popularity in the aquaculture industry. *M. nipponense* is widely distributed in the freshwater and low-salinity estuarine regions across the whole of China, with an annual production of 226,392 tons in 2023, producing huge economic benefits of more than USD 20 billion [11]. A previous study reported that the LC_50_ values of alkalinity tolerance in juvenile Taihu No2 (a new variety of *M. nipponense* through genetic selection by a freshwater fisheries research center) for 12 h, 24 h, 48 h, and 96 h were 27.66 mmol/L, 26.94 mmol/L, 22.51 mmol/L, 15.00 mmol/L, and 14.42 mmol/L, with a safety value of 4.71 mmol/L, under conditions of a water temperature of 23.1 ± 1.48 °C, a pH of 8.9 ± 0.30, a salinity of 0.62 ± 0.27, and a dissolved oxygen level of 7.2 ± 0.30 mg/L [12]. The main regions for *M. nipponense* aquaculture were in the southern and eastern parts of China, including Jiangsu province, Anhui province, Zhejiang province, and Jiangxi province, of which the annual production was more than 200 thousand tons. A reasonable explanation for this is that the water resources of the northeastern and northwestern parts of China were mainly saline–alkaline water and that the alkalinity tolerance of this species is insufficient to adapt and survive in such water environments. Previous studies have reported that high saline and alkali levels have stressful effects on the survival of organisms in water bodies. Thus, it is urgently needed to explore the mechanism of alkalinity adaption in *M. nipponense*, including the identification of key genes involved in the process of alkalinity adaption, which play essential roles in the genetic improvement of alkalinity tolerance in this species.

The gill is an important organ in aquatic animals, which mainly functions to absorb oxygen from the water environment. The surface of the gill filaments is covered by the blood vessels, and these vessels promote the absorption of oxygen from the water environment into the blood for respiratory function [13,14]. In the present study, we aimed to analyze the acute effects of alkali exposure on the gills of *M. nipponense* through performing histological observations, the measurement of antioxidant enzymes, and transcriptome profiling analysis under an alkali concentration of 10 mmol/L for 0, 1, 4, and 7 days. The present study provided valuable evidence for the analysis of alkali tolerance in *M. nipponense*, promoting the aquaculture of this species in saline and alkali regions.

## 2. Results

### 2.1. Changes in Antioxidant Enzymes Caused by Alkali Treatment

**Figure 1:**
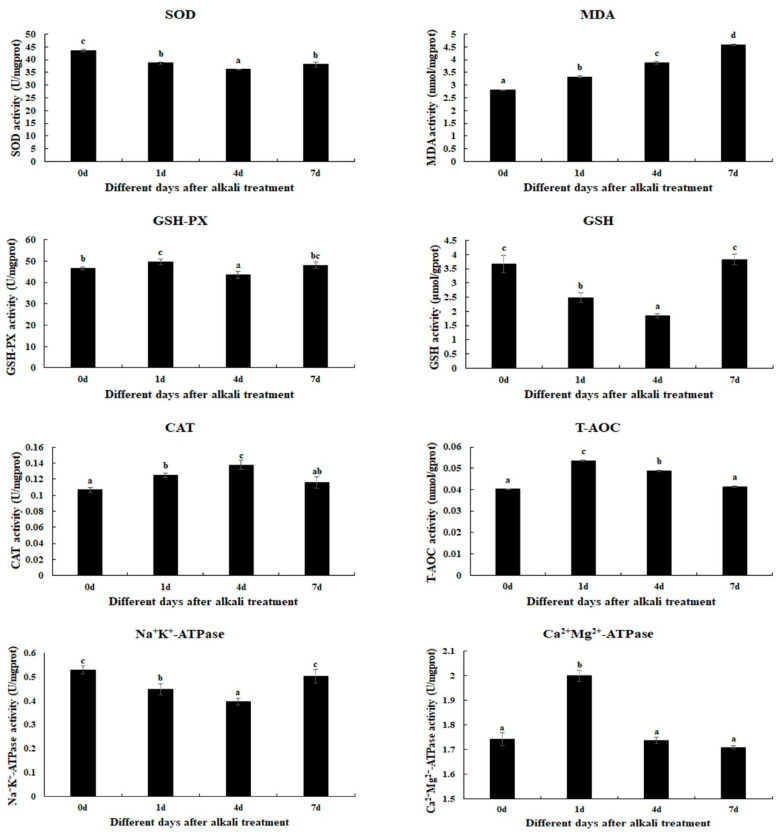
The measurements of the activities of antioxidant enzymes in the gills after different days of alkalinity exposure under the concentration of 10 mmol/L. Data are shown as the mean ± SD (standard deviation) of tissues from three biological replicates. Letters indicate significant differences in the activities of antioxidant enzymes between different days after alkalinity exposure. CAT: catalase; GSH: glutathione; GSH-PX: glutathione peroxidase; MDA: malondialdehyde; SOD: superoxide dismutase; T-AOC: total antioxidant capacity.

The changes in antioxidant enzyme activities are determined in gills after 1 day, 4 days, and 7 days under alkali treatment of 10 mmol/L (Figure 1). The alkali concentration of 10 mmol/L stimulates the activities of MDA, GSH-PX, CAT, T-AOC, and Ca^2+^Mg^2+^-ATPase. The MDA activities are increased with the increase in treatment time (*p* < 0.05). The activities of GSH-PX, T-AOC, and Ca^2+^Mg^2+^-ATPase reach a peak at Day 1 after alkali treatment (*p* < 0.05), while the CAT activity reaches a peak at Day 4 after alkali treatment (*p* < 0.05). The activities of GSH and Na^+^K^+^-ATPase gradually decrease at Day 1 and Day 4 after alkali treatment (*p* < 0.05), and then increase to the normal level at Day 7 (*p* > 0.05). However, the SOD activities are observed to decrease after alkali treatment (*p* < 0.05).

### 2.2. Morphological Changes in Gills Caused by Alkali Treatment

**Figure 2:**
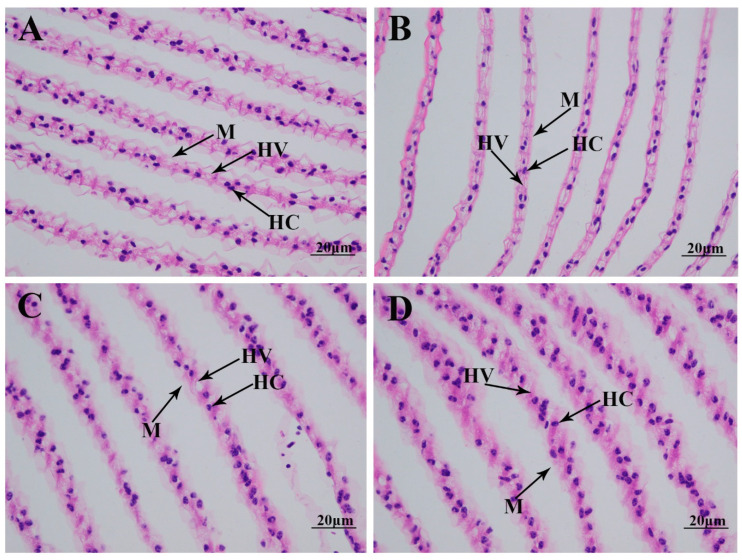
The morphological changes in the gills under an alkalinity of 10 mmol/L. HC: hemocytes; HV: hemolymph vessel; M: membrane. Scale bars = 20 µm. (**A**) morphology of gills without alkalinity exposure; (**B**) morphology of gills after 1 day of alkalinity exposure; (**C**) morphology of gills after 4 days of alkalinity exposure; (**D**) morphology of gills after 7 days of alkalinity exposure.

The morphological changes in the gills under alkali treatment of 10 mmol/L are determined using hematoxylin and eosin (HE) staining (Figure 2). Histological observation of the normal gill reveals that the normal gill comprises hemocytes, hemolymph vessels, and membrane. A short time of alkali treatment (1 day) does not result in significant damage to the gill morphology. However, the membrane is observed to be loosened from Day 4 after alkali treatment, resulting in an increase in hemolymph vessels and affecting the normal respiratory function of the gills [15,16].

### 2.3. Changes in Gene Expressions in Gills Caused by Alkali Treatment

Transcriptome profiling analysis is used to identify the differentially expressed genes (DEGs) in the gills after 1 day, 4 days, and 7 days under alkali treatment of 10 mmol/L. The DEGs are selected, using the criterion of >2.0 for an up-regulated gene and <0.5 for a down-regulated gene. The present study aims to analyze the continuous changes in gene expression in the gills of *M. nipponense* caused by alkalinity exposure. A total of 71 DEGs (59 up-regulated DEGs and 12 down-regulated DEGs), 50 DEGs (6 up-regulated DEGs and 44 down-regulated DEGs), and 488 DEGs (301 up-regulated DEGs and 187 down-regulated DEGs) are selected between G0 and G1, G1 and G4, and G4 and G7, respectively.

**Figure 3:**
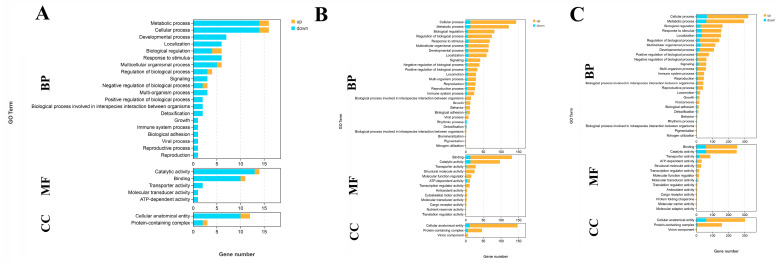
GO analysis of DEGs in the gills after different days of alkalinity exposure under a concentration of 10 mmol/L. (**A**) GO analysis between G0 and G1. (**B**) GO analysis between G1 and G4. (**C**) GO analysis between G4 and G7. BP: biological process; MF: molecular function; CC: cellular component.

A total of 45 DEGs, 40 DEGs, and 327 DEGs are annotated in the GO database among the comparison of G0 vs. G1, G1 vs. G4, and G4 vs. G7, respectively. Cellular process, metabolic process, catalytic activity, binding, and cellular anatomical entity represented the main enriched functional groups in all of these three comparisons (Figure 3).

**Figure 4:**
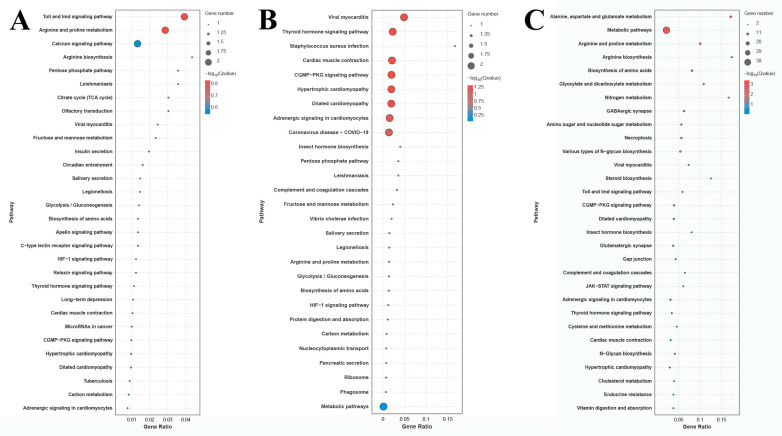
KEGG analysis of DEGs in the gills after different days of alkalinity exposure under a concentration of 10 mmol/L. (**A**) KEGG analysis between G0 and G1. (**B**) KEGG analysis between G1 and G4. (**C**) KEGG analysis between G4 and G7.

The number of DEGs in the comparison of G0 vs. G1, G1 vs. G4, and G4 vs. G7 is 12, 10, and 72 DEGs, respectively (Figure 4). A total of 155 metabolic pathways are identified in the comparison of G4 vs. G7, and the number of DEGs in each metabolic pathway ranges from 1 to 38. The main enriched metabolic pathways in the comparison of G4 vs. G7 include metabolic pathways, biosynthesis of amino acids, amino sugar and nucleotide sugar metabolism, glycolysis/gluconeogenesis, lysosomes, and phagosomes.

### 2.4. Identification of Candidate Genes Involved in the Alkaline Acclimation of M. nipponense

A total of 10 DEGs were selected from the above metabolic pathways, based on the fold change in gene expression, which were considered as strong candidate genes involved in the regulation of alkaline acclimation in *M. nipponense* (Table 1). Fructose-bisphosphate aldolase was differentially expressed in all three comparisons. In addition, the other nine DEGs were differentially expressed between G4 and G7, of which eight were up-regulated at Day 7 after alkalinity exposure.

### 2.5. qPCR Verification

**Figure 5:**
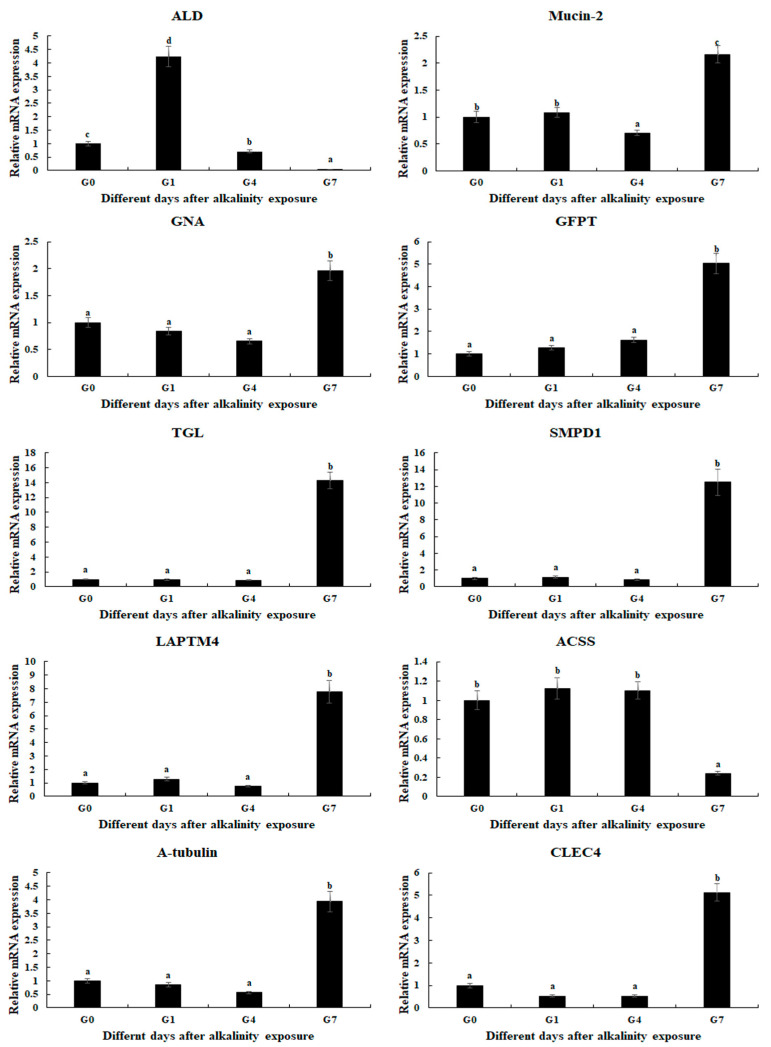
Verification of DEGs’ expression in the gills after different days of alkaline treatment of 10 mmol/L by qPCR analyses. Data are shown as the mean ± SD (standard deviation) of tissues from three biological replicates. Letters indicate a significant difference between different days.

qPCR analysis was used to verify the expression of selected DEGs, which were consistent with those of RNA-Seq (Figure 5). The relative expressions of DEGs at G0 were set as 1, and the expressions at the other time points were compared with G0. Fructose-bisphosphate aldolase (*ALD*) was differentially expressed in all three comparisons, which reached a peak after 1 day of alkalinity exposure (*p* < 0.05). In addition, the expressions of eight DEGs were significantly up-regulated at Day 7 after alkalinity exposure, including Mucin-2, Glucosamine 6-phosphate N-acetyltransferase (*GNA*), Glutamine-fructose-6-phosphate transaminase (*GFPT*), Gastric triacylglycerol lipase (*TGL*), Sphingomyelin phosphodiesterase 1 (*SMPD1*), Lysosomal-associated transmembrane protein 4 (*LAPTM4*), *CLEC4*, and α-tubulin.

## 3. Discussion

The safe alkali value for *M. nipponense* is only 4.71 mmol/L, which is dramatically lower than that of fish species, leading to low annual production in saline–alkaline water regions [12]. Thus, a new variety for *M. nipponense* is urgently needed that has a strong ability to resist alkali treatment. Gills function to absorb oxygen from the water environment, playing essential roles for aquatic animals in the resistance of environmental stress [17]. The present study identifies the effects of alkalinity exposure on the gills of *M. nipponense*, including the selection of candidate genes from the gills involved in the regulation of alkaline acclimation, promoting understanding of the alkaline response in *M. nipponense*.

The measurement of antioxidant enzymes plays essential roles in analyzing the effects on the behavior of prawns under environmental stress [18,19]. Aquatic animals will experience varying degrees of stress response when they suffer environmental changes, leading to the production of a large amount of active free radicals in the body [20,21]. The excessive accumulation of free radicals in aquatic animals leads to tissue damage, the disruption of physiological functions, and a reduction in immune function [22]. Previous studies have reported that the activities of antioxidant enzymes will be stimulated in order to maintain the dynamic balance of free radical content in the body of aquatic animals under alkali treatment. For example, a carbonate alkalinity of 15 mmol/L resulted in a decrease in the SOD and T-AOC activities of the liver and intestine of *Cyprinus carpio* Songpu, while stimulating MDA activity [23]. Alkali concentrations of 32 mmol/L and 64 mmol/L resulted in a significant increase in SOD activities in the liver of *G. przewalskii* after 3 days of treatment [24]. The activities of SOD and CAT were stimulated in the liver of *Triplophysa dalaica* when the fish were exposed to alkalinity [25]. A previous study indicated that alkali treatment stimulated the activities of MDA, GSH, and GSH-PX in the gills of *M. nipponense* [26]. The present study indicates that the activities of MDA, GSH-PX, CAT, T-AOC, and Ca^2+^Mg^2+^-ATPase are stimulated under an alkali concentration of 10 mmol/L, predicting these antioxidant enzymes play essential roles in the removal of active free radicals from *M. nipponense*, in order to protect the prawns from the damage of active free radicals.

A previous study indicated that the ions HCO_3_^−^, CO_3_^2−^, and OH^−^ in saline–alkaline water resources can directly act on the surface epithelial cells of the gills of aquatic animals, disrupting the normal functions of the Cl^−^ and HCO_3_^−^ ion-exchange system on the outer surface of gill cuticle cells. This results in organic damage to the gill tissue, leading to an imbalance in the acid–base buffering system [14,17]. Morphological changes caused by alkali treatment have been widely identified in aquatic animals. No obvious damage was observed in the gills of *L. waleckii* with a strong alkali tolerance under a water environment with a high alkali concentration, while a high alkali concentration resulted in the fusion and detachment of gill cells in *L. waleckii* with a low alkali tolerance, leading to the loss of physiological function [27]. A water environment with high saline–alkaline concentrations resulted in changes in the morphological structures of gills in *G. przewalskii*, in order to adapt to changes in the water environment [28,29,30]. The phenomenon of the deformation and fracture of gill fragments was observed in *Eriocheir sinensis* after alkali treatment [31]. A previous study identified morphological changes in the gills of *M. nipponense* caused by alkali treatment at different concentrations after 96 h. A low alkali concentration (<4 mmol/L) did not result in obvious morphological damage to the gills in *M. nipponense*, while a high alkali concentration (>8 mmol/L) was observed to destroy the normal structure of the membrane and hemolymph vessels, thus affecting the respiratory function of the gills [26]. In the present study, alkali treatment destroys the normal structure of the gill membrane after 4 days under an alkali concentration of 10 mmol/L, resulting in an increase in hemolymph vessels, which is consistent with the previous study.

Transcriptome profiling analyses have been widely used to identify the key metabolic pathways and genes in aquatic animals involved in the regulation of adaptive mechanism under a water environment with a high alkali concentration, including *Lateolabrax maculatus* [32], *Luciobarbus capito* [33], and *Leuciscus waleckii* [34,35]. The metabolic pathways involved in the process of stress response and extreme environment adaptation were identified as the main enriched metabolic pathways of DEGs in such analyses, including phenylalanine, tyrosine, and tryptophan biosynthesis, cell cycle, and DNA replication, indicating the physiological processes of immune response and energy metabolism play essential roles in the regulation of alkaline acclimation in aquatic animals [26]. The present study identifies alkali-tolerance-related metabolic pathways and genes from the gills of *M. nipponense* through performing transcriptome profiling analysis under alkali treatment of 10 mmol/L. Significant changes in gene expression were not observed after 4 days of alkali treatment under such a concentration, indicating short-term alkali treatment has limited effects on the changes in gene expression in the gills of *M. nipponense*. Metabolic pathways, biosynthesis of amino acids, amino sugar and nucleotide sugar metabolism, glycolysis/gluconeogenesis, lysosomes, and phagosomes represent the main enriched metabolic pathways between *G4* and *G7* in the present study, predicting these metabolic pathways and DEGs from these metabolic pathways may play essential roles in regulating the alkali response in *M. nipponense*. A previous study has identified alkali-tolerance-related pathways from the gills of *M. nipponense* after alkali treatment for 96 h at different alkali concentrations, and phagosomes, lysosomes, glycolysis/gluconeogenesis, purine metabolism, amino sugar and nucleotide sugar metabolism, and endocytosis represented the main enriched metabolic pathways, which is similar to the present study.

Lysosomes are membrane-bound organelles, which are widely identified in eukaryotic cells. Lysosomes contain many hydrolytic enzymes, playing a crucial role in cellular digestion and waste disposal. Lysosomes mainly function in the digestion of substances that enter the cell from outside and the digestion of the local cytoplasm or organelles in the cell. Lysosomes are ruptured to release hydrolytic enzymes in order to digest the entire cell when the cells have aged [36,37]. *TGL*, *SMPD1*, and *LATMP4* were enriched in the metabolic pathway of lysosomes. Triacylglycerols are an important energy and structural substance of wild organisms, including aquatic animals [38,39]. Triacylglycerol lipases are identified as the chief lipases, playing essential roles in the regulation of triacylglycerol catabolism in microorganisms, plants, and animals [40]. An acid-stable gastric lipase catalyzes the hydrolysis of dietary triacylglycerols in the stomach [41]. Triacylglycerol hydrolysis continues in the duodenum by the synergetic actions of gastric and colipase-dependent pancreatic lipases and bile secretion [42]. *SMPDs* catalyze the rapid generation of ceramides through cleaving the phosphocholine group from membrane-bound sphingomyelins, in order to respond to various stimuli [43]. Ceramides are the central intermediates of sphingolipid metabolism and are also considered as signaling molecules, participated in the regulation of stress and pro-inflammatory cytokines [44]. Previous studies identified that mutations in the *SMPD1* gene resulted in the deficiency of lysosomal acid sphingomyelinase activity [45,46]. *LATMPs* are embedded in the membrane of lysosomes, playing essential roles in maintaining the normal functions of lysosomes. *LATMPs* are organelles responsible for breaking down and recycling cellular waste and debris [47]. A previous study identified that the overexpression of *LATMP4s* may promote the potential proliferation and/or detoxification of cells, contributing to cancerous transformation or maintenance [48].

The internal defense mechanisms of aquatic animals include humoral defense through antimicrobial peptides and cellular defense through phagocytosis and encapsulation by hemocytes [49,50]. Hemocytes engulf pathogens into vesicles during phagocytosis that are called phagosomes. Phagosomes undergo acidification, playing essential roles in the elimination of pathogens via lysosomal enzymes. Phagosome acidification is identified as a crucial defense mechanism against intracellular pathogens [50]. *CLEC4* and α-tubulin were significantly up-regulated at Day 7 after alkalinity exposure, indicating their potential roles in the regulation of alkaline acclimation in *M. nipponense*. The detyrosination of α-tubulin is considered as a crucial regulatory signal, playing essential roles in the regulation of mitosis and muscle mechanotransduction [51]. The dysregulation of α-tubulin detyrosination results in various pathological states, including increased tumor aggressiveness [52], the onset of neuronal disorders [53], heart failure [54], and cardiomyopathy [55]. The C-type lectin is a non-specific humoral immune factor, which can recognize the saccharide structures on the surface of a pathogen via its carbohydrate recognition domain when a pathogen invades an aquatic animal, subsequently triggering its specific binding to the pathogen [56]. C-type lectins play essential roles in the immune response in aquatic animals through regulating various physiological processes, including pattern recognition, the aggregation of blood cells and bacteria, activation of the complement system, and the enhancement of phagocytosis as an opsonin [57]. Lysosomes and phagosomes are immune-related metabolic pathways, predicting that DEGs from these two metabolic pathways participate in the regulation of alkaline acclimation through enhancing the initial immune response of *M. nipponense*, in order to protect the prawns from the damage of alkalinity exposure.

The biosynthesis of amino acids is a complex process regulated by multiple enzymatic reactions and metabolic pathways. Amino acids are the fundamental building blocks of proteins, playing indispensable roles in numerous biological processes, including cellular growth, tissue repair, and metabolic regulation [58]. *ALD* is differentially expressed in all three comparisons, and it is enriched in the metabolic pathways of both biosynthesis of amino acids and glycolysis/gluconeogenesis, predicting it plays essential roles in the regulation of alkaline acclimation in *M. nipponense*. *ALD* is a class of aldolase which can function as an enzyme, participating in carbohydrate metabolism in the Calvin–Benson cycle, gluconeogenesis, and glycolysis. In addition, *ALD* can also be considered as a non-enzymatic protein, involving in protein binding, gene transcription, and signal transduction [59,60,61].

Glycolysis/gluconeogenesis is an important metabolic pathway in organisms, involved in the regulation of energy production [62]. Glycolysis is a process participating in the conversion of glucose into pyruvate when oxygen is absent. This process catalyzes the production of small amounts of ATP and NADH (reducing power) [63]. Glycogenesis is an important physiological process of converting the non-sugar substances (certain amino acids, lactate, pyruvate, and glycerol) into glucose or glycogen, catalyzed by enzymes in the liver and kidneys, in order to maintain a stable glucose level. Gluconeogenesis is considered the reverse process of glycolysis. During this process, two pyruvate molecules are constructed into one glucose molecule, occurring under conditions with sufficient oxygen [64]. Acetyl-CoA is an important metabolite of intermediary metabolism which has multiple functions. It is considered as a substrate of the tricarboxylic acid cycle, as well as a key precursor of lipid synthesis, and a donor of the acetyl group for protein and histone acetylation [65]. Acetyl-CoA synthetases (*ACSSs*) promote the conversion of acetate into acetyl-CoA [66]. *ACSS2* can stimulate the production of acetyl-CoA, promoting malignant cell growth and survival in some solid tumors, under conditions of hypoxia, glucose deprivation, and low serum [67,68,69].

The products of amino sugar and nucleotide sugar metabolism have many physiological functions in living organisms. These products play essential roles in cells, especially the role of maintaining and repairing the cell wall. Amino sugars are important substances for energy metabolism produced through glucose metabolism. Amino sugars are involved in regulation of the signal transduction and cell recognition within cells [70,71]. Nucleotide sugars widely exist in cells. Nucleotide sugars are important metabolic products, playing essential roles in the regulation of signal transduction, cell division, and cell apoptosis [72,73]. Chitin is an important structure, promoting the formation of the cuticle in insects, which acts as a barrier against pathogens [74]. *GNA* is a key enzyme regulating chitin biosynthesis in insects, promoting chitin formation [75]. *GFPT* catalyzes the formation of glucosamine 6-phosphate, which is the first and rate-limiting step for protein glycosylation and chitin synthesis [76]. The biosynthesis of amino acids, glycolysis/gluconeogenesis, and amino sugar and nucleotide sugar metabolism are energy metabolism-related metabolic pathways, promoting energy production. Thus, DEGs from these metabolic pathways are predicted to participate in the alkaline acclimation of *M. nipponense* through providing energy.

The qPCR verifications of DEGs are consistent with those of RNA-Seq, indicating the accuracy of the present study. *ALD* is differentially expressed in all three comparisons. The other genes show no difference from Day 0 to Day 4, while eight genes are up-regulated at Day 7 after alkalinity exposure.

## 4. Materials and Methods

### 4.1. Tissue Collection

A total of 150 healthy prawns with a body weight of 3.62 ± 0.79 g were provided by the Dapu *M. nipponense* Breeding Base in Wuxi, China (120°13′44″ E, 31°28′22″ N), and maintained in lab conditions with a water temperature of 26.0 °C ± 1.2, alkalinity of 0.8 mmol/L, and dissolved oxygen of >6.0 mg/L, prior to 3 days of alkali treatment. The water environment with an alkali concentration of 10 mmol/L was prepared by adding NaHCO_3_ under conditions of a water temperature of 26.0 °C ± 1.2, pH = (7.81–8.32) and a dissolved oxygen level of >6.0 mg/L. The alkali concentrations were measured according to the criterion SC/T9406-2012 [77]. The prawns were treated in the water environment with an alkali concentration of 10 mmol/L, and the gills were collected at 0 (control, G0), 1 day (G1), 4 days (G4), and 7 days (G7) after alkali treatment. Three gills were collected and stayed in 4% paraformaldehyde until the histological slicing and observation. Five gills were collected and pooled at together to form a biological replicate. Three biological replicates were prepared for transcriptome profiling analysis, qPCR, and assessment of changes in antioxidant enzymes. The tissue samples were immediately stored at −80 °C after collection, in order to prevent the degradation of RNA, and stayed under such conditions until experimental analysis.

### 4.2. Measurement of the Activities of Antioxidant Enzymes

The commercial kit from Nanjing Jiancheng Bioengineering Institute was used to measure the changes in antioxidant enzymes in gills after acute treatment at an alkali concentration of 10 mmol/L. The measured antioxidant enzymes in the present study include total antioxidant capacity (T-AOC), superoxide dismutase (SOD), malondialdehyde (MAD), catalase (CAT), glutathione (GSH), glutathione peroxidase (GSH-PX), Ca^2+^Mg^2+^-ATPase, and Na^+^K^+^-ATPase. A microplate reader was used to measure all of the antioxidant indexes (Bio-rad iMark, San Francisco, CA, USA), following the manufacturer’s instructions.

### 4.3. Histological Observation

Hematoxylin and eosin (HE) staining was used to measure the morphological changes in the gills after alkali treatment with a concentration of 10 mmol/L. Three collected gills from each treated time point were sliced (three biological replicates), and two slices were prepared from each gill (two technical replicates). The detailed procedures of HE staining have been well described in previous studies [78,79]. Briefly, the collected gills are first dehydrated using different concentrations of ethanol, and then different percentages of a xylene/wax mixture are used to make the dehydrated gills transparent and embed them. The slicer is used to slice the embedded gills up to a 5 µm thickness (Leica, Wetzlar, Germany), and HE is finally used to stain the slices for 3–8 min. Slides are observed using the Olympus SZX16 microscope (Olympus Corporation, Tokyo, Japan).

### 4.4. Transcriptome Profiling Analysis

The changes in gene expression caused by the alkali treatment were selected through transcriptome profiling analysis performed on the Illumina Hiseq-2500 sequencing platform (Illumina, CA, USA). The RNAiso Plus Reagent (TaKaRa, Shiga, Japan) was employed to extract the total RNA from the gills of each biological replicate, following the manufacturer’s instructions. The total RNA concentration was measured by a spectrophotometer (Eppendorf, Hamburg, Germany), and the integrity of the extracted total RNA was measured by a 2100 Bioanalyzer (Agilent Technologies, Inc., Santa Clara, CA, USA) with an RNA integrity number (RIN) value of >7.0. The detailed procedures for the RNA-Seq and bio08520information analysis have been well described in previous published papers [80,81]. Briefly, a total of 4 µg of total RNA is used to construct the library, and the Illumina Hiseq-2500 sequencing platform is used to conduct the sequencing under the sequencing strategy PE150.

Fastp software (version 0.20.0) is employed to remove the low-quality raw reads with the default parameters [82]. The HISAT2 software (version 0.20.0) is then employed to map the obtained clean reads to the *M. nipponense* reference genome (Genbank access numbers: GCA_015104395.2) [83]. Genes are annotated in the Gene Ontology (GO) (http://www.geneontology.org/, accessed on 14 September 2024) [84], Cluster of Orthologous Groups (COG) (http://www.ncbi.nlm.nih.gov/COG/, accessed on 14 September 2024) [85], and Kyoto Encyclopedia of Genes and Genomes (KEGG) databases (http://www.genome.jp/kegg/, accessed on 14 September 2024) [86], using an E-value of 10^−5^ [80]. Gene expression is calculated using the FPKM method, where FPKM = cDNA fragments/mapped fragments (millions)/transcript length (kb), using HTSeq-count (version 0.6.1) [87]. DESeq2 (version 0.6.1) is used to perform the differential expression analysis [88]. The Benjamini–Hochberg correction method is used to calculate the false discovery rate (FDR) [89] with q-value < 0.05. A fold change > 2.0 is considered to determine up-regulated differentially expressed genes (DEGs), and a fold change < 0.5 is considered to determine down-regulated DEGs.

### 4.5. qPCR Analysis

qPCR is employed to measure the difference in the gene expression of DEGs between each time point under a water environment with an alkalinity of 10 mmol/L, in order to verify the accuracy of RNA-Seq. The detailed procedures of qPCR analysis have been well described in previous studies [90,91]. The commercial kit from the Shanghai Sangon Company (Shanghai, China, UNlQ-10 Column Trizol Total RNA Isolation Kit) is used to extract the total RNA from the gills at each time point. The concentration of extracted total RNA is measured using a spectrophotometer (Eppendorf, Hamburg, Germany), and the integrity of the total RNA is measured using a 1.2% agarose gel. The cDNA template is synthesized from 1 μg total RNA using the commercial kit (PrimeScript™ RT reagent kit, Takara Bio Inc., Shiga, Japan), following the manufacturer’s instructions. The expression level is measured by the UltraSYBR Mixture (CWBIO, Beijing, China), following the reaction system in the manufacturer’s instructions. The qPCR analysis is conducted on the Bio-Rad iCycler iQ5 Real-Time PCR System (Bio-Rad, Hercules, CA, USA), which can carry out the SYBR Green RT-qPCR assay. All primers used for the qPCR analysis are listed in Table 2. The eukaryotic translation initiation factor 5A (*EIF*) is used as the reference gene for qPCR analysis in the present study, which has been proven to be stably expressed under various conditions in *M. nipponense* [92]. The 2^−ΔΔCT^ method is used to determine the relative expression levels [93].

### 4.6. Statistical Analysis

The statistical analysis of gene expression and antioxidant enzyme activities is conducted using SPSS Statistics 23.0, estimated by one-way ANOVA, followed by Duncan’s multiple range test [90,91]. Homogeneity of variance tests and normality analysis were performed, prior to the statistics analysis, in order to ensure the accuracy of statistical analysis. *p* < 0.05 indicates significance. Quantitative data are expressed as the mean ± SD.

## 5. Conclusions

In conclusion, alkaline treatment stimulates the activities of MDA, GSH-PX, CAT, T-AOC, and Ca^2+^Mg^2+^-ATPase and results in damage of the hemolymph vessels under an alkalinity of 10 mmol/L. In addition, their gene expressions remain stable after 4 days of alkalinity exposure. Furthermore, metabolic pathways, biosynthesis of amino acids, amino sugar and nucleotide sugar metabolism, lysosomes, glycolysis/gluconeogenesis, and phagosomes represent the main enriched metabolic pathways of DEGs between Day 4 and Day 7, indicating the processes of immune response and energy metabolism play essential roles in the response to alkalinity exposure in *M. nipponense*. The present study will dramatically promote the survival rate and aquaculture of this species under water environments with high alkalinity.

## Figures and Tables

**Table 1:** The candidate genes involved in the regulation of alkaline acclimation.

Gene	Accession Number	Metabolic Pathway	Log2 (Fold Change)		
			S0 vs. S1	S1 vs. S4	S4 vs. S7
Fructose-bisphosphate aldolase-like (*ALD*)	ncbi_135219851	Biosynthesis of amino acids, glycolysis/gluconeogenesis	5.4	−6.9	−5.3
Mucin-2	ncbi_135210831	Amino sugar and nucleotide sugar metabolism			2.7
Glucosamine 6-phosphate N-acetyltransferase (*GNA*)	ncbi_135213629	Amino sugar and nucleotide sugar metabolism			1.7
Glutamine—fructose-6-phosphate transaminase (*GFPT*)	ncbi_135226562	Amino sugar and nucleotide sugar metabolism			1.4
Gastric triacylglycerol lipase (*TGL*)	ncbi_135218763	Lysosome			4.0
Sphingomyelin phosphodiesterase 1 (*SMPD1*)	ncbi_135213205	Lysosome			3.2
Lysosomal-associated transmembrane protein 4 (*LAPTM4*)	ncbi_135223659	Lysosome			2.0
Acetyl-CoA synthetase 2 (*ACSS2*)	ncbi_135226390	Glycolysis/gluconeogenesis			−1.3
A-tubulin	ncbi_135198818	Phagosome			1.5
C-type lectin domain family 4 (*CLEC4*)	ncbi_135199509	Phagosome			2.7

**Table 2:** The primers used in the present study.

Gene	Primer
*ALD*	F: TACCCACGACTTGGAACGTG
	R: CTTTGCAACCTGTGCAGCAT
*Mucin-2*	F: AGCAAGGTCTCGTGTTGCTT
	R: TTACTCTTGTTCTGCGCCGT
*GNA*	F: AAAGCTGGACTGGTCGAAGG
	R: TGTCTCCCACTTTGGTCAGC
*GFPT*	F: AGTTGGAAGGTGCCTTTGCT
	R: ATCGTGCAAGACCGTGTGAT
*TGL*	F: CTAATCGGCAGAAACGCAGC
	R: CGCTCTCTTAGGCTGACTCG
*SMPD*	F: GCCAGTGTGTCGAGGAATCA
	R: AGGGATGTTCACGCTCCAAG
*LAPTM4*	F: TGGGACTGGGAACCGATACT
	R: CCATCACGTTTGGTCCTTGC
*ACSS2*	F: TTCCCCCATTTGCGCACTTA
	R: CCGTCTGGAAACCACCTGAA
*A-tubulin*	F: AACACGTCCCAAGAGCTGTC
	R: AGTGTCCACGGGCATAGTTG
*CLEC4*	F: ATGGGAGTCACGTCAGAGGA
	R: TTCCCGTGGTAAAGGACTGC
*EIF*	F: CATGGATGTACCTGTGGTGAAAC
	R: CATGGATGTACCTGTGGTGAAAC

## Data Availability

The raw data of the present study have been submitted to NCBI with the accession number SRX28124957-SRX28124968.
